# Economic burden of moderate to severe irritable bowel syndrome with constipation in six European countries

**DOI:** 10.1186/s12876-019-0985-1

**Published:** 2019-05-07

**Authors:** Jan Tack, Vincenzo Stanghellini, Fermín Mearin, Yan Yiannakou, Peter Layer, Benoit Coffin, Magnus Simren, Jonathan Mackinnon, Gwen Wiseman, Anne Marciniak, on behalf of the IBIS-C Study group, Bouchoucha, Ducrotte, Macaigne, Mion, Schneider, Le Sidanier, Tardy, Zerbib Andresen, Bischoff, Jander, Jung, Thomas Jung, Krammer, Langhorst, Omankowsky, S. Omankowsky, Schiefke, Steuer, Van der Voort, Martin Luther Krankenhausbetrieb, Von Arnim, Annese, Cuomo, De Angelis, Di Mario, Di Stefano, Matteo, Ferrini, Gasbarrini, Giaccobe, Marchi, Marzio, Repici, Savarino, Angós, Berdier, Brotons, Caballero-Plasencia, Caballero, Clavé, Fort, Josep Trueta, García, Gómez, Martínez, Perelló, Rey, Sánchez-Antolín, Serra, Tantinà, Torán, Ekesbo, Hellström, Kjellström, Lindberg, Walter, Arebi, Bridger, Butt, Emmanuel, Eugenicos, Kaushik, Millar, Sanders, Whorwell

**Affiliations:** 1 University Hospital Gasthuisberg, Leuven, Belgium; 2 University Hospital S.Orsola, Bologna, Italy; 3 Centro Médico Teknon, Barcelona, Spain; 4 County Durham and Darlington NHS Trust, Durham, UK; 5 Israelitic Hospital, Hamburg, Germany; 6 AP-HP Louis-Mourier Hospital, Colombes, and Paris Diderot University, Paris, France; 7 Sahlgrenska University Hospital, Göteborg, Sweden; 8 TFS Develop S.L, Barcelona, Spain; 9 Allergan International, Marlow, UK; 10 University of Leuven, Herestraat 49, 3000 Leuven, Belgium

**Keywords:** IBS, IBS-C, Economic analysis, Healthcare resource utilisation, Europe

## Abstract

**Background:**

Irritable bowel syndrome with predominant constipation (IBS-C) is a complex disorder with gastrointestinal and nervous system components. The study aim was to assess the economic burden of moderate to severe IBS-C in six European countries (France, Germany, Italy, Spain, Sweden and the UK).

**Methods:**

An observational, one year retrospective-prospective (6 months each) study of patients diagnosed in the last five years with IBS-C (Rome III criteria) and moderate to severe disease at inclusion (IBS Symptom Severity Scale score ≥ 175). The primary objective was to assess the direct cost to European healthcare systems.

**Results:**

Five hundred twenty-five patients were included, 60% (range: 43.1–78.8%) suffered from severe IBS-C. During follow-up 11.1–24.0% of patients had a hospitalisation/emergency room (ER) visit, median stay range: 1.5–12.0 days and 41.1–90.4% took prescription drugs for IBS-C. 21.4–50.8% of employed patients took sick leave (mean: 11.6–64.1 days). The mean annual direct cost to the healthcare systems was €937.1- €2108.0. The total direct cost (combined costs to healthcare systems and patient) for IBS-C was €1421.7–€2487.1.

**Conclusions:**

IBS-C is not a life-threatening condition; however, it has large impact on healthcare systems and society. Direct and indirect costs for moderate to severe IBS-C were high with the largest direct cost driver being hospitalisations/ER visits.

## Background

Irritable bowel syndrome (IBS) is a chronic functional bowel disorder (FBD) characterised by symptoms of recurrent abdominal pain accompanied by altered bowel function [1]. The prevalence of IBS varies between region and country, however pooled prevalence estimates IBS to occur in 5–20% of the global population [2]. The Rome criteria [3] are the global standards developed by experts in functional bowel disorders which enable physicians to identify and diagnose disorders such as IBS. Rome characterises IBS into subtypes based on the predominant bowel habit: IBS with predominant diarrhoea (IBS-D), IBS with predominant constipation (IBS-C), IBS with mixed bowel habits (IBS-M), and IBS unclassified (IBS-U) [3]. Nevertheless, the clinical continuum of symptoms has been shown to result in short-term subtype instability with around 40% of patients alternating between subtypes on a monthly basis [4, 5].

Influence by the nervous system adds to the clinical complexity of this FBD as it results in fluctuating symptoms, both temporally (waxing and waning cycle of high disease activity and remission) and by severity [6, 7]. Yet despite severity fluctuations, moderate-severe IBS is estimated to account for around 60% of all IBS cases and has been shown to impose a considerable burden on patients [8]. This burden is manifested into a health economic burden through direct medical costs to healthcare systems (HS) and indirect costs related to work absenteeism or work impairment. Studies suggest that the burden of illness for IBS is quite substantial with all IBS subtypes requiring similar levels of healthcare resource utilisation [9–14].

Continued research and re-evaluation of diagnostic criteria have greatly improved physicians’ ability to differentiate IBS from other FBDs and enhance the accuracy of subtype classification [3, 15]. For example, IBS-C and chronic constipation are often confused due to their similarity in defecation patterns, despite IBS-C symptomatology being dominated by abdominal pain [1, 16]. It is estimated that IBS-C accounts for around 30% of IBS cases [17]. Treatment revolves around the use of therapies which are not specifically approved for treating IBS-C, such as laxatives, antispasmodics, prokinetics, and bulking agents (e.g. dietary fibres). Although useful for treating constipation, in some patients these therapies show poor efficacy, tolerability and are unable to treat all key IBS-C symptoms when used individually [18, 19]. In Europe, the guanylate cyclase-C agonist linaclotide is the only pharmacological treatment approved for the treatment of IBS-C and has been shown to be cost-effective compared to antidepressants [20, 21].

Given the symptom and severity complexities of IBS-C there is a notable paucity of information related to health economic burden and resource utilisation, especially in sufferers at the more severe end of the severity spectrum. Newer agents approved to treat IBS-C may potentially decrease the need for frequent visits to physicians and multiple medications, possibly translating into lower healthcare utilisation and drug-related costs. Therefore, this study aimed to learn more about the burden of the disease in a real-world population of moderate to severe patients with IBS-C across six European countries.

## Methods

The IBIS-C study was an observational, 12 month retrospective-prospective (6 months each) multicentre study conducted in six European countries: France, Germany, Italy, Spain, Sweden, and the UK. The first patient was included in April 2012 and the last patient last visit was in January 2014. Patients were recruited from primary or specialist care.

Screening was performed using retrospective data from patient records. Patients who met the eligibility criteria were included. Patients were included in the study if they were ≥ 18 years of age, diagnosed with IBS-C in the last 5 years using the Rome III criteria (recurrent IBS pain or discomfort present for at least three days per month in the last three months; ≥ 2 of the following: improvement with defecation, onset associated with a change in stool frequency, or a change in stool form; ≥25% of bowel movements being hard or lumpy stools; < 25% of bowel movements being loose or watery stools), and had moderate to severe IBS-C at inclusion: defined as an Irritable Bowel Syndrome Symptom Severity Scale (IBS-SSS) score ≥ 175 (moderate severity: ≥175–300; severe: > 300) [22]. Patients were excluded if they had participated in a clinical trial involving an experimental IBS-C treatment in the six months prior to starting the observational period, or they had any condition that, in the investigator’s opinion, would impact the patient’s ability to complete the study. This study was conducted in accordance with the Declaration of Helsinki as well as in compliance with ICH good clinical practices guidelines. The following ethics committees approved the trial protocol and its amendments: Comité Consultatif sur le Traitement de l’Information en matière de Recherche dans le domaine de la Santé (Paris, France), Israelitisches Krankenhaus (Hamburg, Germany), University of Bologna (Bologna, Italy), Humanitas Hospital IRCS (Milano, Italy), Floraspe Renzetti Hospital (Lanciano, Italy), Agostino Gemelli University Hospital (Rome, Italy), University of Parma (Parma, Italy), University of Pisa (Pisa, Italy), University of Pescara (Pescara, Italy), Careggi University (Firence, Italy), University of Napoli Federico II (Napoli, Italy), University of L’Aquila (L’Aquila, Italy), University of Messina (Messina, Italy), University of Genoa (Genoa, Italy). Ospedale *S. Maria* di Ca′ Foncello (Treviso, Italy), Fondazione IRCCS Policlinico San Matteo (Pavia, Italy), Centro médico Teknon (Barcelona, Spain), Hospital Clínico San Carlos (Madrid, Spain), Hospital Universitario 12 de Octubre (Madrid, Spain), Hospital Germans Trias I Pujol (Badalona, Spain), Hospital de Bellvitge (Barcelona, Spain), Consorci Sanitari del Maresme (Mataró, Spain), Hospital Universitario Virgen de la Macarena (Sevilla, Spain), Hospital Universitari Doctor Josep Trueta (Girona, Spain), Hospital Universitari Sant Joan de Reus (Reus, Spain), National Institute for Health Research (London, UK).

**Fig. 1:**
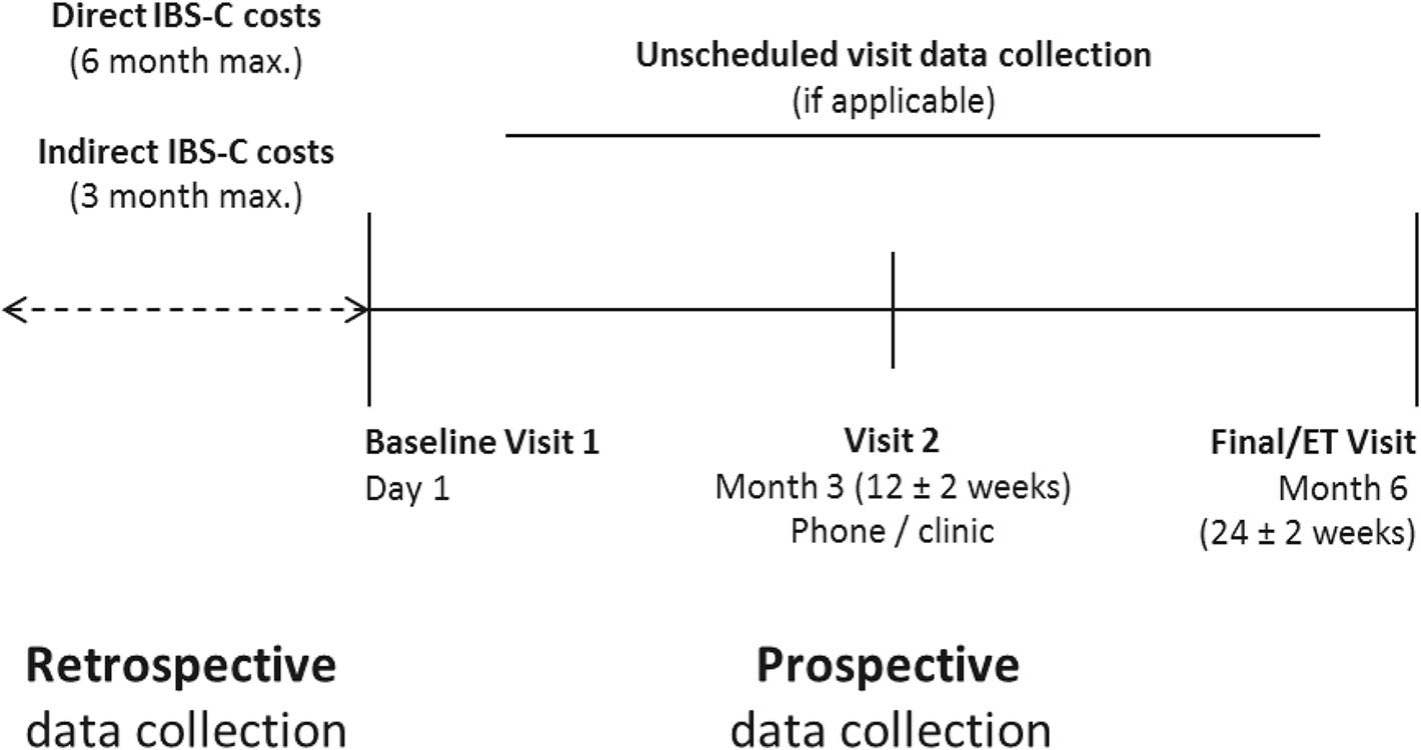
Study design

The study design is shown in Fig. 1. Baseline and 6-month retrospective data from included patients were obtained from patient medical records and patient interviews. Demographic and clinical data were collected at baseline. Symptom severity (IBS-SSS) was collected at baseline and 6 months. Healthcare resource utilisation directly related to IBS-C (general and specialist medical consultations, hospitalisations, diagnostic tests, therapies, management of adverse reactions) was collected via a questionnaire that specified whether the costs were public or private. Healthcare resource data were collected at baseline for up to 6 months for direct costs retrospectively and 3 months for indirect costs. Three-month indirect costs were multiplied by two to provide a retrospective 6-month estimate of indirect costs associated with IBS-C. Prospective healthcare resource utilisation data were collected during routine follow-up at 3 months (± 0.5 months) and 6 months (± 1 month) / early termination. All healthcare resource utilisation data were calculated for patients that used the resource.

Direct costs were calculated both from a country healthcare system’s perspective and the patient’s perspective using unit costs for each country. Hospitalisation costs were estimated from national diagnosis related group (DRG) databases. For the patient’s perspective, only the sum of non-prescription medication, complementary therapies and HS-related medications, consultations, hospitalisations, and diagnostic procedures that were paid for by the patient were taken into account; private consultation and private diagnostic procedure costs were not included.

Productivity losses in the week prior to baseline and at the 6-month visit were collected using the work productivity and activity impairment questionnaire for IBS-C (WPAI:IBS-C): a four component score for absenteeism, presenteeism (reduced productivity while at work), overall work impairment, and daily activity impairment (impairment in activities performed outside of work) [23]. Absenteeism, presenteeism, and overall work impairment were recorded for employed patients and daily activity impairment for all patients. Indirect costs were calculated using questions about sick leave and work productivity impairment. The indirect costs were: cost of productivity for sick leave (total number of days lost * 1 days’ salary), cost for work impairment (total number of work hours lost * 1 h’ salary). In the event that the salary value was missing the mean value of the salary reported by employed patients was converted to gross income and used to estimate indirect costs.

Exploratory analyses were performed and no confirmatory statistical tests were performed. Demographics, baseline characteristics, healthcare resource utilisation characteristics and productivity losses were summarised using descriptive statistics based on non-missing observations. Costs were calculated as a mean with 95% confidence interval (95% CI; calculated using 1000 bootstrap samples). Swedish krona (SEK) and Pound Sterling (GBP) were converted post hoc into Euro (EUR) using exchange rates of 1 SEK = 0.1134 EUR and 1 GBP = 1.2025 EUR, respectively.

A sample size of 90 patients per country was calculated to produce a 95% CI equal to the sample mean ± ≤ 20% of the standard deviation of the direct costs associated with IBS-C [12]. For each country the retrospective, prospective, and combined data periods were analysed separately. The separate analyses were then compared to determine whether there was any statistical difference. An analysis of the combined data is presented for patient demographics and baseline characteristics; healthcare resource utilisation, work productivity and activity impairment, and all costs are presented by country.

## Results

A total of 525 patients across all countries were included in the study (France: *N* = 59; Germany: *N* = 102; Italy: *N* = 112; Spain: N = 112; Sweden: *N* = 36; UK: *N* = 104) between April 2012 and January 2014. Over follow-up there were 62 (11.8%) discontinuations; 36 (6.9%) lost to follow-up, 19 (3.6%) with data missing, 5 (1.0%) withdrawals at the patients’ personal request, and 2 (0.4%) withdrawals due to illness.

### Demographic, clinical and lifestyle characteristics at baseline

Table 1 shows the main characteristics of this study cohort. In summary, 459 (87.4%) of patients were included from specialist care with France, Italy and UK including 100% of patients from specialist care (range: 60.8–100%). Patients were predominantly female (86.9%), with a mean ± standard deviation (SD) age of 45.3 ± 15.8 years old. A total of 135 (25.7%) patients (range: 16.7–39.0%) had a prior appendectomy/cholecystectomy; seven (1.3%) patients (range: 1.0–3.4%) had bariatric surgery or surgery to remove a gastrointestinal (GI) tract segment; and 127 (24.2%) patients (range: 14.3–35.6%) had another type of surgery of the abdomen, pelvis, or retroperitoneal structures. IBS-C symptoms were present for a mean duration of 12.8 ± 13.1 years (range: 9.6–15.6 years). Patients were diagnosed with IBS-C for on average 3.0 ± 5.2 years (range: 2.3–4.6 years) prior to study inclusion.

**Table 1:** Patient Demographics

	FRANCE	GERMANY	ITALY	SPAIN	SWEDEN	UK	TOTAL
*N*	*59*	*102*	*112*	*112*	*36*	*104*	*525*
Study centre recruitment, n (%)							
Primary Care	0 (0.0)	40 (39.2)	0 (0.0)	25 (22.3)	1 (2.8)	0 (0.0)	66 (12.6)
Specialist Care	59 (100.0)	62 (60.8)	112 (100.0)	87(77.7)	35 (97.2)	104 (100.0)	459 (87.4)
Age (years), mean (SD)	47.7 (15.7)	47.6 (18.1)	41.7 (17.0)	46.8 (13.7)	41.4 (12.7)	45.5 (14.6)	45.3 (15.8)
Female, n (%)	49 (83.1)	85 (83.3)	96 (85.7)	96 (85.7)	33 (91.7)	97 (93.3)	456 (86.9)
Higher education (university or similar), n (%)	16 (27.1)	42 (41.2)	50 (44.6)	30 (26.8)	15 (41.7)	31 (29.8)	184 (35.0)
Employment status, n (%)							
Unemployed	12 (20.3)	9 (8.8)	15 (13.4)	31 (27.7)	4 (11.1)	25 (24.0)	96 (18.3)
Student	1 (1.7)	10 (9.8)	15 (13.4)	3 (2.7)	2 (5.6)	1 (1.0)	32 (6.1)
Part time (≤20 h/ week)	2 (3.4)	12 (11.8)	8 (7.1)	10 (8.9)	3 (8.3)	18 (17.3)	53 (10.1)
Full time (> 20 h/ week)	29 (49.2)	44 (43.1)	62 (55.4)	49 (43.8)	24 (66.7)	47 (45.2)	255 (48.6)
Retired	14 (23.7)	27 (26.5)	12 (10.7)	19 (17.0)	3 (8.3)	13 (12.5)	88 (16.8)
Employed patients with salary above the national average^a^, n (%)	11 (19.0)	19 (18.6)	23 (20.5)	42 (37.5)	20 (55.6)	38 (36.5)	153 (29.2)
Level of physical exercise^b^, n (%)							
Low	38 (64.4)	44 (43.1)	68 (60.7)	67 (59.8)	11 (30.6)	53 (51.0)	281 (53.5)
Intermediate	15 (25.4)	47 (46.1)	34 (30.4)	33 (29.5)	14 (38.9)	38 (36.5)	181 (34.5)
High	2 (3.4)	11 (10.8)	10 (8.9)	12 (10.7)	11 (30.6)	13 (12.5)	59 (11.2)
Consumes alcohol, n (%)	6 (10.2)	31 (30.4)	25 (22.3)	22 (19.6)	27 (75.0)	61 (58.7)	172 (32.8)
Current smoker, n (%)	8 (13.6)	15 (14.7)	22 (19.6)	26 (23.2)	7 (19.4)	22 (21.2)	100 (19.0)
Patients who follow a diet, n (%)	12 (20.3)	21 (20.6)	50 (44.6)	48 (42.9)	15 (41.7)	43 (41.3)	189 (36.0)
Type of diet, n (%)							
Hypocaloric	2 (3.4)	2 (2.0)	7 (6.3)	10 (8.9)	0 (0.0)	3 (2.9)	24 (4.6)
Low sodium	1 (1.7)	1 (1.0)	2 (1.8)	9 (8.0)	1 (2.8)	1 (1.0)	15 (2.9)
Low carbohydrate	1 (1.7)	1 (1.0)	2 (1.8)	7 (6.3)	4 (11.1)	5 (4.8)	20 (3.8)
High-fibre	4 (6.8)	4 (3.9)	24 (21.4)	27 (24.1)	4 (11.1)	9 (8.7)	72 (13.7)
Low-fibre	0 (0.0)	2 (2.0)	6 (5.4)	4 (3.6)	1 (2.8)	12 (11.5)	25 (4.8)
Other	5 (8.5)	13 (12.7)	14 (12.5)	6 (5.4)	7 (19.4)	18 (17.3)	63 (12.0)
Previous GI surgery, n (%)							
Appendectomy/cholecystectomy	23 (39.0)	31 (30.4)	25 (22.3)	20 (17.9)	6 (16.7)	30 (28.8)	135 (25.7)
Bariatric surgery or surgery to remove a GI tract segment	2 (3.4)	3 (2.9)	0 (0.0)	0 (0.0)	1 (2.8)	1 (1.0)	7 (1.3)
Other surgeries of the abdomen, pelvis, or retroperitoneal structures	17 (28.8)	19 (18.6)	16 (14.3)	27 (24.1)	11 (30.6)	37 (35.6)	127 (24.2)
Time since IBS-C diagnosis (years), mean (SD)	2.6 (6.3)	4.6 (8.4)	2.9 (3.8)	2.3 (2.8)	2.3 (3.4)	2.6 (4.0)	3.0 (5.2)
Symptom duration (years), mean (SD)	13.5 (13.5)^1^	15.0 (16.9)	10.4 (9.1)	9.6 (9.9)	15.6 (12.2)	15.3 (14.9)	12.8 (13.1)

^a^Average gross income (€/year): France: 35,511; Germany: 42,633; Italy: 26,040; Spain: 27,674; Sweden: 40,568; UK: 39,303. ^b^Low level: Sports activities 0–1 times a week/ walk less than 0.5 h per day; Medium level: Sports activities 2–3 times per week/ walk at least 0.5 h per day; High level: sports activities at least 4 times per week. ^1^N = 51

SD Standard deviation; GI gastrointestinal

In this cohort of moderate to severe IBS-C patients, on average 60.0% had severe IBS-C at baseline (range: 43.1–78.8%), 38.5% had moderate IBS-C (range: 20.2–56.9%), and 1.5% had missing data (Table 2). Using the IBS-SSS questionnaire, current abdominal pain and distention were reported for 95.8% (range: 89.8–98.1%) and 92.8% (range: 88.1–96.2%) of patients, respectively. Furthermore, abdominal pain was reported to occur for 6.0 ± 2.9 out of every 10 days on average (range: 5.4–7.0) and the most severe IBS symptoms were a dissatisfaction with bowel habits and interference with life in general which had mean scores of 76.6 ± 22.0 (range: 69.0–82.9) and 70.7 ± 21.8 (range: 59.6–79.5) respectively.

**Table 2:** IBS-C symptoms at baseline

	FRANCE	GERMANY	ITALY	SPAIN	SWEDEN	UK	TOTAL
*N*	*59*	*102*	*112*	*112*	*36*	*104*	*525*
IBS-SSS categorical items, n (%)							
Current abdominal pain	53 (89.8)	99 (97.1)	108 (96.4)	107 (95.5)	34 (94.4)	102 (98.1)	503 (95.8)
Current abdominal distention	52 (88.1)	91 (89.2)	106 (94.6)	104 (92.9)	34 (94.4)	100 (96.2)	487 (92.8)
IBS-SSS score (SD)							
Severity of abdominal pain^a^	57.5 (25.3)	49.3 (23.8)	59.8 (19.8)	56.5 (21.4)^3^	56.6 (21.0)^4^	71.7 (20.6)	59.1 (22.9)
Number of days with abdominal pain^b^	7.0 (2.9)	5.4 (2.8)	5.4 (2.8)	5.7 (2.8)	6.8 (3.0)^5^	6.9 (3.0)	6.0 (2.9)
Severity of abdominal distention^a^	66.5 (23.6)^1^	60.0 (21.4)^2^	64.8 (20.8)	65.0 (23.6)	66.6 (19.8)	75.2 (19.5)	66.4 (22.0)
Dissatisfaction with bowel habits^a^	71.6 (23.3)	69.0 (23.7)	78.9 (20.2)	77.9 (19.6)	75.8 (23.6)	82.9 (20.9)	76.6 (22.0)
Interference with life in general^a^	79.5 (15.9)	68.2 (19.0)	59.6 (25.5)	71.4 (21.0)	70.9 (16.0)	79.4 (19.9)	70.7 (21.8)
IBS-SSS overall score ^c^	338.9 (78.6)	288.3 (78.8)	311.1 (75.1)	315.4 (82.2)	317.3 (81.8)	373.1 (82.5)	323.2 (84.3)
Categorical severity of IBS-C, n (%)							
Mild (< 175)	0 (0.0)	0 (0.0)	0 (0.0)	0 (0.0)	0 (0.0)	0 (0.0)	0 (0.0)
Moderate (175 ≤ 300)	17 (28.8)	58 (56.9)	43 (38.4)	47 (42.0)	16 (44.4)	21 (20.2)	202 (38.5)
Severe (> 300)	37 (62.7)	44 (43.1)	68 (60.7)	64 (57.1)	20 (55.6)	82 (78.8)	315 (60.0)

^a^0–100; best to worst. ^b^in every10 days. ^c^0–500; best to worst. ^1^N = 52. ^2^N = 91. ^3^N = 99. ^4^N = 30. ^5^N = 32

*IBS-SSS* Irritable Bowel Syndrome Symptom Severity Scale, *SD* Standard Deviation

### Healthcare resource utilisation related to IBS-C

During the 12-month retrospective- prospective study the majority of patients visited a General Practitioner (GP) (73.5%; range: 58.9–88.4%) and/or a specialist (92.2%; range: 79.4–100.0%) (Table 3). The mean number of visits to physicians under the healthcare systems was higher for GPs (range: 2.1–6.4) than gastroenterologists (range: 1.7–4.0). Overall Sweden had the lowest mean (95% CI) number of GP and gastroenterologist visits, 2.1 (1.4, 2.7) and 1.7 (1.4, 2.0) respectively, in comparison to Italy which had the highest mean number of visits 6.4 (4.0, 8.7) and 4.0 (3.1, 5.0) respectively. By contrast, private consultations were less frequent, yet maintained a similar GP: gastroenterologist ratio of visits. In the UK, the proportion of patients seeking private consultation visits to either GPs or gastroenterologists was very low in comparison to Germany, France and Spain.

**Table 3:** Healthcare resource utilisation

	FRANCE	GERMANY	ITALY	SPAIN	SWEDEN	UK
*N*	*59*	*102*	*112*	*112*	*36*	*104*
Medical consultations (HS + Private), n (%)						
GP	45 (76.3)	80 (78.4)	66 (58.9)	99 (88.4)	22 (61.1)	74 (71.2)
Specialists	59 (100.0)	81 (79.4)	112 (100.0)	94 (83.9)	34 (94.4)	104 (100.0)
*A) HS consultations*						
Gastroenterologist, n (%)	59 (100.0)	51 (50.0)	112 (100.0)	67 (59.8)	23 (63.9)	104 (100.0)
Mean visits (95% CI)^a^	2.2 (1.8, 2.6)	2.0 (1.5, 2.4)	4.0 (3.1, 5.0)	2.3 (1.9, 2.7)	1.7 (1.4, 2.0)	2.7 (2.3, 3.0)
GP, n (%)	21 (35.6)	41 (40.2)	65 (58.0)	93 (83.0)	21 (58.3)	73 (70.2)
Mean visits (95% CI)^a^	4.2 (2.4, 6.0)	4.3 (3.2, 5.5)	6.4 (4.0, 8.7)	3.8 (2.9, 4.7)	2.1 (1.4, 2.7)	6.2 (4.2, 8.3)
*B) Private consultations*						
Gastroenterologist, n (%)	19 (32.2)	46 (45.1)	33 (29.5)	47 (42.0)	12 (33.3)	4 (3.8)
Mean visits (95% CI)^a^	1.9 (1.1, 2.7)	2.7 (1.7, 3.7)	2.1 (1.6, 2.6)	2.2 (1.6, 2.8)	2.3 (1.4, 3.1)	2.0 (0.0, 4.3)
GP, n (%)	29 (49.2)	68 (66.7)	4 (3.6)	17 (15.2)	2 (5.6)	2 (1.9)
Mean visits (95% CI)^a^	3.9 (2.7, 5.0)	4.2 (3.2, 5.2)	3.1 (0.0, 6.4)	3.6 (2.3, 4.9)	1.5 (0.0, 7.9)	5.0 (0.0, 55.8)
Hospitalisations or emergency room visits						
Any visit, n (%)	10 (16.9)	19 (18.6)	15 (13.4)	22 (19.6)	4 (11.1)	25 (24.0)
Number of hospitalisations, mean (95% CI)^b^	1.5 (0.9, 2.0)	2.4 (1.3, 3.4)	1.8 (0.7, 2.9)	1.3 (1.0, 1.6)	2.5 (0.0, 5.3)	1.7 (1.2, 2.3)
Median	1.0	1.0	1.0	1.0	2.1	1.0
Number of days hospitalised, mean (95% CI)^b^	5.7 (0.0, 12.2)	19.7 (3.2, 36.2)	17.9 (0.0, 41.6)	1.5 (0.6, 2.4)	10.7 (0.0,28.3)	11.8 (2.5, 21.1)
Median	3.1	9.0	6.0	1.5	12.0	6.0
Diagnostic tests						
Any test n (%)	34 (57.6)	68 (66.7)	83 (74.1)	65 (58.0)	22 (61.1)	54 (51.9)
Number of tests, mean (95% CI)^b^	3.4 (2.4, 4.3)	4.0 (3.4, 4.6)	4.5 (3.9, 5.2)	4.1 (3.4, 4.7)	3.5 (2.5, 4.6)	3.6 (2.9, 4.4)
Median	2.5	3.5	4.0	4.0	3.0	3.0
Pharmacological therapies, n (%)						
Prescription drug	31 (52.5)	73 (71.6)	54 (48.2)	95 (84.8)	33 (91.7)	97 (93.3)
Prescription drug for IBS-C	30 (50.8)	56 (54.9)	46 (41.1)	89 (79.5)	28 (77.8)	94 (90.4)
Non-prescription drug for IBS-C	36 (61.0)	71 (69.6)	92 (82.1)	63 (56.3)	25 (69.4)	66 (63.5)
Complementary therapies, n (%)	18 (30.5)	28 (27.5)	41 (36.6)	33 (29.5)	16 (44.4)	37 (35.6)
Absenteeism and work impairment questionnaire for employed patients (over one year)						
Any sick leave taken, n (%)	12 (38.7)	19 (33.9)	15 (21.4)	14 (23.7)	12 (44.4)	33 (50.8)
Number of times on sick leave, mean (95% CI)^c^	3.3 (1.0, 5.6)	4.5 (3.0, 6.0)	6.9 (3.5, 10.2)	6.3 (1.9, 10.6)	5.7 (0.8, 10.6)	5.2 (3.8, 6.6)
Number of days on sick leave, mean (95% CI)^c^	64.1 (17.0, 111.2)	29.5 (10.5, 48.5)	11.6 (4.1, 19.2)	52.4 (0.0, 114.4)	51.3 (0.0, 113.6)	25.9 (12.7, 39.1)
Any work impairment while working, n (%)	16 (51.6)	37 (66.1)	18 (25.7)	31 (52.5)	19 (70.4)	53 (81.5)
Number of hours of work impairment, mean (95% CI)^c^	69.1 (22.0, 116.1)	140.4 (88.8, 192.0)	83.2 (18.4, 148.0)	54.8 (34.0, 75.7)	280.3 (146.5, 414.1)	161.9 (103.6, 220.2)

^a^Number of visits (calculated in patients with at least 1 visit)

^b^Calculated in patients with at least one hospitalisation/diagnostic test

^c^In patients with at least one sick leave.

*95% CI* 95% Confidence Interval, *GP* General practitioner/family doctor, *HS* Healthcare system

The proportion of patients requiring hospitalisation or emergency room visit for IBS-C showed variability across Europe, with the lowest reported in Sweden (11.1%) and the highest in the UK (24.0%). The median number of days hospitalised ranged from 1.5 days in Spain to 12.0 days in Sweden. The most frequently reported diagnostic related group related to hospitalisation was “gastroenteritis and/or abdominal pain”.

Diagnostic tests related to IBS-C were performed on 51.9% (UK) to 74.1% (Italy) of patients. On average, the most common diagnostics test were blood tests (both haematology [37.9%] and clinical chemistry [33.0%]), abdominal ultrasounds (21.7%), and colonoscopies (17.5%) (Table 4). On average, prescription and non-prescription drugs used to treat IBS-C was similar; 41.1% (Italy) to 90.4% (UK) took prescription drugs and 56.3% (Spain) to 82.1% (Italy) took non-prescription drugs. The most commonly prescribed drugs across countries were: macrogol plus electrolytes (21.2%); prucalopride (16.4%); *Plantago ovata* (11.5% [not prescribed in Italy]); and mebeverine (10.2% [not prescribed in Sweden]).

**Table 4:** Procedures, investigations or tests over 12 months due to IBS-C

Procedures, investigations or tests due to IBS-C, n (%)	FRANCE	GERMANY	ITALY	SPAIN	SWEDEN	UK	TOTAL
*N*	*59*	*102*	*112*	*112*	*36*	*104*	*525*
Anascopy	3 (5.1)	3 (2.9)	0 (0.0)	0 (0.0)	0 (0.0)	0 (0.0)	6 (1.1)
Anorectal manometry	7 (11.9)	0 (0.0)	10 (8.9)	14 (12.5)	5 (13.9)	5 (4.8)	41 (7.8)
Antibody test tissue	2 (3.4)	2 (2.0)	17 (15.2)	7 (6.3)	3 (8.3)	3 (2.9)	34 (6.5)
Antibody testing endomysial	0 (0.0)	1 (1.0)	14 (12.5)	3 (2.7)	2 (5.6)	0 (0.0)	20 (3.8)
Barium enema	0 (0.0)	0 (0.0)	0 (0.0)	5 (4.5)	0 (0.0)	5 (4.8)	10 (1.9)
Blood tests, clinical chemistry	11 (18.6)	40 (39.2)	42 (37.5)	38 (33.9)	12 (33.3)	30 (28.8)	173 (33.0)
Blood tests, haematology	14 (23.7)	45 (44.1)	58 (51.8)	42 (37.5)	10 (27.8)	30 (28.8)	199 (37.9)
Colonoscopy	12 (20.3)	22 (21.6)	23 (20.5)	18 (16.1)	4 (11.1)	13 (12.5)	92 (17.5)
Computed tomography, abdominal	5 (8.5)	1 (1.0)	4 (3.6)	6 (5.4)	2 (5.6)	8 (7.7)	26 (5.0)
C-reactive protein (CRP)	8 (13.6)	22 (21.6)	13 (11.6)	6 (5.4)	8 (22.2)	8 (7.7)	65 (12.4)
Endoscopy, Small intestine	0 (0.0)	2 (2.0)	0 (0.0)	0 (0.0)	2 (5.6)	1 (1.0)	5 (1.0)
Endoscopy, Upper GI	6 (10.2)	8 (7.8)	7 (6.3)	6 (5.4)	1 (2.8)	5 (4.8)	33 (6.3)
Erythrocyte sedimentation test	5 (8.5)	5 (4.9)	16 (14.3)	14 (12.5)	2 (5.6)	4 (3.8)	46 (8.8)
Esophagoscopy	3 (5.1)	7 (6.9)	6 (5.4)	5 (4.5)	2 (5.6)	1 (1.0)	24 (4.6)
Faecal occult blood	0 (0.0)	4 (3.9)	5 (4.5)	4 (3.6)	3 (8.3)	0 (0.0)	16 (3.0)
Faecal ova and parasite test	0 (0.0)	1 (1.0)	12 (10.7)	4 (3.6)	0 (0.0)	2 (1.9)	19 (3.6)
Fistulogram	0 (0.0)	0 (0.0)	0 (0.0)	0 (0.0)	0 (0.0)	1 (1.0)	1 (0.2)
Hydrogen breath test	5 (8.5)	6 (5.9)	10 (8.9)	8 (7.1)	0 (0.0)	0 (0.0)	29 (5.5)
Magnetic resonance imaging	7 (11.9)	6 (5.9)	4 (3.6)	4 (3.6)	2 (5.6)	3 (2.9)	26 (5.0)
Microbiological tests	1 (1.7)	4 (3.9)	4 (3.6)	1 (0.9)	0 (0.0)	1 (1.0)	11 (2.1)
Radiology, Abdominal	2 (3.4)	2 (2.0)	13 (11.6)	8 (7.1)	0 (0.0)	9 (8.7)	34 (6.5)
Radiology, Upper GI	0 (0.0)	0 (0.0)	4 (3.6)	1 (0.9)	0 (0.0)	2 (1.9)	7 (1.3)
Sigmoidoscopy/proctosigmoidoscopy	0 (0.0)	4 (3.9)	1 (0.9)	2 (1.8)	2 (5.6)	6 (5.8)	15 (2.9)
Small bowel aspiration	0 (0.0)	1 (1.0)	1 (0.9)	0 (0.0)	1 (2.8)	0 (0.0)	3 (0.6)
Stool examination	3 (5.1)	15 (14.7)	14 (12.5)	5 (4.5)	2 (5.6)	1 (1.0)	40 (7.6)
Thyroid function test	3 (5.1)	7 (6.9)	20 (17.9)	18 (16.1)	3 (8.3)	11 (10.6)	62 (11.8)
Ultrasound, abdominal	8 (13.6)	36 (35.3)	46 (41.1)	16 (14.3)	1 (2.8)	7 (6.7)	114 (21.7)
Urinalysis	1 (1.7)	22 (21.6)	23 (20.5)	18 (16.1)	4 (11.1)	9 (8.7)	77 (14.7)
Other	9 (15.3)	6 (5.9)	6 (5.4)	10 (8.9)	7 (19.4)	19 (18.3)	57 (10.9)

*GI* gastrointestinal

### Work productivity and activity impairment

During the 6 month prospective follow-up, at least one period of sick leave was taken by 21.4% (Italy) to 50.8% (UK) of patients. Whilst on sick leave, the mean number of days on leave varied substantially across countries; from 11.6 days in Italy to 64.1 days in France. Over the course of the year, patients had 3.3 separate episodes of leave in France to 6.9 episodes in Italy. For those who reported work impairment while working, wide variability was seen again with 25.7% of patients reporting impairment in Italy to 81.5% of patients in the UK.

According to the WPAI:IBS-C questionnaire, in the week prior to inclusion in the study the percentage of work productivity loss in employed patients was high; between 27.7% (Spain) and 51.5% (UK) (Table 5). In addition, absenteeism and presenteeism for employed patients varied from 3.1–18.5% and 27.6–47.9%, respectively. The overall daily activity impairment varied from 36.3–56.8%.

**Table 5:** Impairment in work productivity in employed patients (WPAI:IBS-C questionnaire) and daily activities in all patients with moderate to severe IBS-C at baseline and 6 months

		FRANCE	GERMANY	ITALY	SPAIN	SWEDEN	UK
	*Total*	*59*	*102*	*112*	*112*	*36*	*104*
Presenteeism, % ^a^	*N*	*27*	*52*	*66*	*45*	*24*	*58*
	Baseline	44.4 (32.9)	35.6 (28.2)	27.6 (27.7)	29.2 (27.5)	41.3 (22.1)	47.9 (28.7)
	*N*	*15*	*49*	*65*	*49*	*20*	*41*
	6 Months	43.3 (27.7)	32.2 (24.9)	25.4 (21.4)	27.1 (23.9)	34.0 (21.4)	38.0 (28.0)
Absenteeism, %^a^	*N*	*21*	*43*	*63*	*46*	*22*	*51*
	Baseline	18.5 (34.9)	14.7 (28.2)	3.1 (9.3)	6.1 (15.8)	6.9 (12.4)	8.4 (24.2)
	*N*	*9*	*33*	*60*	*49*	*18*	*38*
	6 Months	0.9 (2.8)	3.2 (9.1)	0.9 (3.7)	2.9 (5.0)	2.7 (6.7)	7.0 (22.6)
Overall work productivity loss, %^a^	*N*	*21*	*43*	*62*	*50*	*22*	*49*
	Baseline	46.3 (33.2)	44.1 (32.7)	27.7 (28.6)	32.3 (27.2)	44.4 (24.9)	51.5 (27.2)
	*N*	*9*	*33*	*60*	*55*	*18*	*37*
	6 Months	39.6 (29.3)	32.0 (25.7)	26.1 (22.0)	29.8 (24.0)	32.4 (20.2)	39.1 (27.2)
Daily activity impairment, %	*N*	*52*	*95*	*110*	*104*	*35*	*99*
	Baseline	48.1 (29.0)	36.3 (24.8)	41.1 (29.1)	39.6 (27.2)	48.0 (25.4)	56.8 (29.6)
	*N*	*37*	*92*	*105*	*97*	*30*	*72*
	6 Months	50.5 (28.2)	38.0 (23.3)	33.0 (25.1)	37.9 (26.3)	43.0 (28.4)	51.4 (31.7)

^a^Employed patients. All values are presented as mean (SD)

In the week prior to the 6-month visit, absenteeism (0.9–7.0%) was found to have the greatest improvement, whereas work productivity loss (26.1–39.6%) and presenteeism (25.4–43.3%) were found to slightly improve. There was no improvement in the daily activity impairment (33.0–51.4%).

### Direct and indirect costs

**Fig. 2:**
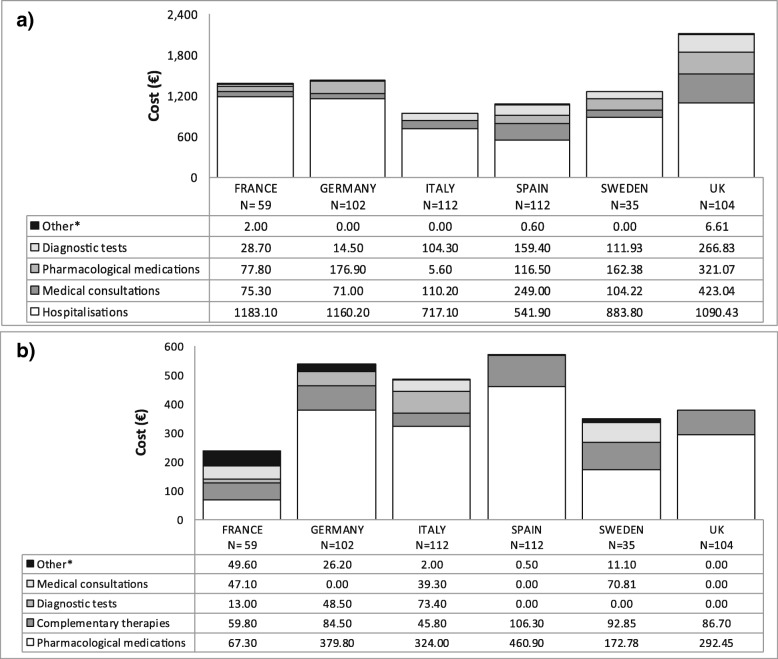
Mean healthcare resource utilisation costs for the (**a**) HS and (**b**) patient. * includes hospitalisations and adverse reactions

Costs related to the management of moderate to severe IBS-C varied greatly between countries. The largest cost for all healthcare systems in the management of IBS-C was hospitalisation (range: € 541.9 € 1183.1) [Fig. 2]. The next largest cost for healthcare systems was medical consultations followed by medication costs (both prescription and non-prescription, and diagnostic tests (Fig. 2a). The largest costs to the patient were medication costs and complementary therapy costs (Fig. 2b).

Overall, the UK had the highest the mean (95% CI) direct cost to the healthcare system at € 2108.0 (€ 1504.1, € 2775.5) while Italy had the lowest overall direct cost at € 937.1 (€ 524.8, € 1528.1). In terms of the direct costs to the patient, France reported the lowest patient costs at € 236.8 (€ 161.7, € 332.9) and Spain reported the highest costs at € 567.6 (€ 333.1, € 840.7) (Table 6). Overall, total mean (95% CI) direct costs ranged from € 1421.7 (€ 947.1, € 2090.0) in Italy to € 2487.1 (€ 1848.2, € 3150.3) in the UK. Mean (95% CI) patient indirect cost had the largest variation in cost between countries; from €339.0 (€ 182.4, € 517.1) in Italy to € 11,248.5 (€ 4580.2, € 20,192.6) in Sweden.

**Table 6:** Direct and indirect annual costs per patient related to IBS-C

	FRANCE	GERMANY	ITALY	SPAIN	SWEDEN	UK
*N*	*59*	*102*	*112*	*112*	*36*	*104*
Direct cost to HS (€)						
Mean	1366.8	1422.6	937.1	1067.3	1276.9	2108.0
(95% CI)	(504.1, 2565.4)	(621.2, 2600.9)	(524.8, 1528.1)	(729.6, 1446.6)	(491.5, 2368.6)	(1504.1, 2775.5)
Min, Max	19.0–24,079.0	0.0, 47,390.0	0.0, 25,363.0	0.0, 9680.0	0.0, 13,937.7	45.7, 16,773.7
Median	186.0	93.5	107.0	262.5	271.3	870.0
Direct cost to Patient (€)						
Mean	236.8	539.0	484.5	567.6	347.1	379.1
(95% CI)	(161.7, 332.9)	(313.4, 840.9)	(371.6, 627.2)	(333.1, 840.7)	(243.7, 484.0)	(221.6, 579.6)
Min, Max	14.0, 2358.0	0.0, 11,506.0	17.0, 6179.0	0.0, 8092.0	0.0, 1716.0	0.0, 6500.7
Median	147.0	129.0	303.0	103.5	209.6	63.7
TOTAL DIRECT COST (€)						
Mean	**1603.7**	**1961.6**	**1421.7**	**1635.0**	**1623.9**	**2487.1**
(95% CI)	**(715.4, 2815.7)**	**(1063.0, 3142.0)**	**(947.1, 2090.0)**	**(1226.0, 2061.4)**	**(803.4, 2710.9)**	**(1848.2, 3150.3)**
Min-Max	**33.0, 24,097.0**	**0.0, 48,403.0**	**17.0, 28,355.0**	**0.0, 10,782.0**	**29.5, 14,100.0**	**57.7, 16,780.9**
Median	**342.0**	**340.0**	**489.0**	**518.5**	**634.8**	**1070.8**
Indirect cost (€)^1^						
Mean	2473.8	2619.0	339.0	1361.9	11,248.5	4097.2
(95% CI)	(831.9, 4594.1)	(1400.2, 4130.6)	(182.4, 517.1)	(312.5, 2866.4)	(4580.2, 20,192.6)	(2498.7, 5984.4)
Min, Max	0.0, 37,753.0	0.0, 50,997.0	0.0, 5232.0	0.0, 59,630.0	0.0, 101,827.3	0.0, 45,271.7
Median	0.0	0.0	0.0	0.0	442.8	156.3

Currency conversion performed on 11th Feb 2014 (EUR/SEK = 8.8183; EUR/GBP = 0.8316). ^1^France *N* = 57

*HS* Healthcare system

Bold data are summation of Direct costs to HS + Direct costs to patient

### Annual cost stratified by severity

When stratified by baseline IBS-C severity, the mean annual cost to healthcare systems for patients with moderate IBS-C (range: € 314.4 - € 1308.4) was approximately a third of the cost to the healthcare systems compared to patients with severe IBS-C (range: € 961.0 - € 2883.3 (Table 7). Direct costs to the patient were similar regardless of severity: moderate IBS-C range: € 204.8 - € 539.2; severe IBS-C range: € 256.8 - € 887.3 per year. Overall direct costs for IBS-C approximately doubled with increased severity: moderate IBS-C range: € 589.2 - € 1642.4; severe IBS-C range: € 1217.8 - € 3770.7. Indirect costs showed lower costs for moderate IBS-C (range: € 297.7 - € 6710.9) compared to severe IBS-C (range: € 370.2- € 14,878.4).

**Table 7:** Annual costs related to moderate and severe IBS-C

	FRANCE		GERMANY		ITALY		SPAIN		SWEDEN		UK	
	MODERATE	SEVERE	MODERATE	SEVERE	MODERATE	SEVERE	MODERATE	SEVERE	MODERATE	SEVERE	MODERATE	SEVERE
*N*	*17*	*37*	*58*	*44*	*43*	*68*	*47*	*64*	*16*	*20*	*21*	*82*
Direct cost to HS (€)												
Mean (95% CI)	421.4 (134.9, 947.3)	961.0 (206.7, 2162.6)	314.4 (163.1, 498.6)	2883.3 (1060.1, 5569.8)	507.0 (187.4, 902.9)	1219.9 (576.8, 2143.1)	675.5 (387.1, 1098.1)	1371.8 (858.4, 1954.0)	1291.0 (185.5, 3384.9)	1264.9 (363.9, 2554.8)	1308.4 (594.4, 2123.6)	2325.0 (1604.3, 3119.0)
Min, Max	19.0, 3956.0	19.0, 16,761.0	0.0, 3747.0	0.0, 47,390.0	0.0, 5316.0	4.0, 25,363.0	0.0, 6601.0	0.0, 9680.0	0.0, 13,937.7	0.0, 11,657.4	45.7, 6984.1	45.7, 16,773.7
Median	157.0	201.0	46.0	236.5	64.0	160.5	199.0	357.5	156.7	271.4	590.4	898.9
Direct cost to Patient (€)												
Mean (95% CI)	204.8 (105.2, 340.2)	256.8 (157.1, 410.7)	274.7 (158.2, 412.3)	887.3 (417.6, 1498.7)	289.2 (228.9, 348.6)	612.8 (438.1, 851.8)	539.2 (110.0, 1033.3)	596.0 (341.8, 952.6)	351.3 (168.3, 616.6)	343.5 (225.4, 481.8)	325.2 (93.3, 613.4)	396.2 (212.6, 642.0)
Min, Max	14.0, 1034.0	14.0, 2358.0	0.0, 2400.0	0.0, 11,506.0	17.0, 739.0	37.0, 6179.0	0.0, 8092.0	0.0, 7004.0	0.0, 1716.0	17.4, 883.4	0.0, 2409.8	0.0, 6500.7
Median	132.0	164.0	71.0	255.0	256.0	372.5	65.0	149.0	177.8	255.2	72.2	52.9
TOTAL DIRECT COST (€)												
Mean (95% CI)	**626.2 (316.4, 1124.0)**	**1217.8 (405.5, 2446.1)**	**589.2 (384.0, 824.7)**	**3770.7 (1814.4, 6496.7)**	**796.3 (439.3, 1227.0)**	**1832.7 (1115.4, 2727.7)**	**1214.7 (683.4, 1783.1)**	**1967.8 (1355.9, 2651.8)**	**1642.4 (493.7, 3637.4)**	**1608.4 (663.0, 3153.5)**	**1633.6 (850.4, 2508.7)**	**2721.3 (2003.0, 3545.2)**
Min-Max	**33.0, 3978.0**	**33.0, 17,017.0**	**0.0, 3822.0**	**0.0, 48,403.0**	**17.0, 6055.0**	**44.0, 28,355.0**	**0.0, 8245.0**	**41.0, 10,782.0**	**29.5, 14,100.0**	**30.1, 12,175.6**	**57.7, 7221.0**	**57.7, 16,780.9**
Median	**361.0**	**334.0**	**190.0**	**742.5**	**315.0**	**610.0**	**339.0**	**648.5**	**492.6**	**760.2**	**829.7**	**1111.1**
Indirect cost (€)												
Mean (95% CI)	2514.1 (125.6, 7733.3)	2464.9 (586.7, 5157.7)	1279.6 (377.1, 2499.4)	4384.7 (2088.3, 7495.6)	297.7 (39.7, 629.3)	370.2 (169.7, 597.6)	362.0 (146.6, 689.7)	2117.6 (328.8, 4725.8)	6710.9 (453.4, 15,788.3)	14,878.4 (4884.1, 29,930.1)	1364.7 (608.3, 2286.3)	4845.1 (2869.9, 7296.5)
Min, Max	0.0, 37,678.0	0.0, 37,753.0	0.0, 23,714.0	0.0, 50,997.0	0.0, 5232.0	0.0, 4154.0	0.0, 5625.0	0.0, 59,630.0	0.0, 50,979.5	0.0, 101,826.8	0.0, 7015.4	0.0, 45,271.7
Median	0.0	0.0	0.0	64.0	0.0	0.0	0.0	0.0	41.3	2696.4	668.6	93.8

Currency conversion performed on 11th Feb 2014 (EUR/SEK = 8.8183; EUR/GBP = 0.8316)

*HS* Healthcare system

Bold data are summation of Direct costs to HS + Direct costs to patient

## Discussion

Irritable bowel syndrome is a complex FBD that is characterised by a wide variety of symptoms. The increasing recognition of the disorder’s complexity highlights the importance of a more detailed understanding of the impact that this disorder has on society. The aim of this study was to assess the economic impact to healthcare systems of moderate to severe IBS-C in patients from six European countries. In terms of characteristics, the patients in this study had similar sociodemographic values to previous IBS studies [9, 11, 13, 14, 24]. Nevertheless, as this study is focused on a more severe patient subgroup, it is worth noting that within this moderate to severe population a higher incidence of prior abdominal surgery was reported compared to IBS patients in general [25].

Overall, patients in this cohort reported a high frequency of symptoms that led to substantial direct and indirect costs for healthcare systems and society. Despite differences in European healthcare system structures direct costs were similar with hospitalisations/ER visits being the largest cost driver [26–28]. Costs attributable to hospitalisations/ER visits in this study were higher than that reported for IBS patients in general [13, 24, 29]. This finding is related to the predominantly severe IBS-C population of this study and suggests that patients with more severe IBS-C may require more inpatient care. Furthermore, as a consequence of this, the costs for medications and consultations accounted for a smaller percentage of total costs than previously reported [12, 24]. It is highly likely that variability between countries included in this study is due to differences in therapeutic management and reimbursement policies. The somewhat unexpected frequency and duration of hospitalisations/ER visits has not been previously reported in cross-sectional surveys and combined with other health economic data suggests that there is a proportion of IBS-C patients who have uncontrolled illness associated with exceptionally high costs [30–32]. For all countries there was a clear asymmetric distribution of cost that highlights a subset of patients within the included population who required substantial medical attention, enough to make the overall economic cost resemble the US healthcare system costs for IBS-C [24, 31, 33].

In terms of resource utilisation, the proportion of patients who underwent diagnostic tests in this study was comparable to that seen in primary care, signifying that patients with more severe IBS-C were still undergoing a diagnosis of exclusion [34]. Yet, despite multiple tests, the lower overall prescription medication use (compared to IBS patients in general) and high non-prescription/ complementary therapy use suggested an overall dissatisfaction with current prescription medications [33, 35]. These current management practices resulted in a degree of absenteeism that was two to three times greater than that reported for other IBS and IBS-C studies [14, 36, 37].

It is worth noting that like healthcare costs, the current management of the disease also exhibits asymmetry that can be attributed to the presence of a subgroup of “heavy resource users”. This reinforces the probability of treatment management being unable to adequately control symptoms over time.

In relation to the direct cost of IBS-C, indirect costs were more substantial and more variable between countries. For example, Sweden had the highest indirect costs at over € 11,000 per year due to Sweden having the highest percentage of employed patients whose income was above the national average in this study, and one of the highest average gross incomes in Europe. The focus on work productivity and sick leave as an estimate of indirect cost is a limitation of this study as salaries and type of employment are variable between countries. As these variables were not adjusted for in the analyses total direct and indirect costs were not combined.

Stratification by disease severity showed that the total direct cost of IBS-C in patients with severe disease severity (60% of the sample) was approximately double the cost of IBS-C for patients with moderate disease severity with some notable exceptions. Total direct costs in Germany showed the largest disparity with severe IBS-C mean costs approximately 6-fold greater than moderate IBS-C costs. By contrast, the greatest similarity in total direct costs was in Sweden, where both moderate and severe IBS-C patients have a direct cost of around € 1600 per year. Mean indirect costs were substantially greater for patients with severe disease compared to patients with moderate disease, however, costs were skewed by a subset of patients with high costs. As moderate-severe IBS accounts for approximately two thirds of all IBS cases, this indicates a high economic cost to both healthcare systems and society [7, 17].

The main limitations of this study were the incomplete assessment of indirect healthcare costs associated with work productivity and absenteeism, and the potential underestimation of healthcare resource utilisation due to the majority of patients being included from specialist care centres and thus benefitting from more dedicated management. This could have also introduced a referral bias in the sample studied. In addition, the exclusion of private consultation and diagnostic procedure costs may further underestimate direct costs. Furthermore, the retrospective element of the study may be associated with some recall bias, leading to some imprecision surrounding estimates. Lastly, variation in the proportion of severe patients in each country may have translated into increased variation between countries.

## Conclusions

Although IBS-C is not a life-threatening condition, this study has shown that moderate to severe IBS-C has a significant and costly impact on healthcare systems and patients. The absence of a standard of care, combined with an absence in the improvement of work impairment, indicates that symptoms in patients with moderate to severe IBS-C remain uncontrolled. These results highlight the need for a greater understanding of more severe IBS patients, moving away from a “one size fits all” management approach to one that recognises the individual complexity of this FBD and which focuses on treating the patients’ individual symptoms.

## Abbreviations

CI: Confidence interval
DRG: Diagnosis related group
ER: Emergency room
EUR: Euro
FBD: Functional bowel disorder
GBP: Pound Sterling
GI: Gastrointestinal
GP: General Practitioner
HS: Healthcare systems
IBS: Irritable bowel syndrome
IBS-C: Irritable bowel syndrome with predominant constipation
IBS-D: Irritable bowel syndrome with predominant diarrhoea
IBS-M: Irritable bowel syndrome with mixed bowel habits
IBS-SSS: Irritable Bowel Syndrome Symptom Severity Scale
IBS-U: Irritable bowel syndrome unclassified
SD: Standard deviation
SEK: Swedish krona
WPAI:IBS-C: Work productivity and activity impairment questionnaire for IBS-C
